# Distinct Patterns of Host Adherence by *Neisseria gonorrhoeae* Isolated from Experimental Gonorrhea

**DOI:** 10.1155/2021/7865405

**Published:** 2021-05-13

**Authors:** Yingxia He, Song Zhang, Yingmiao Zhang, Bicong Wu, Ying Xue, Chenglin Ye, Qiao Li, Adhiambo Njiri Olivia, John Mambwe Tembo, Hongxiang Chen, Huahua Cai, Tie Chen

**Affiliations:** ^1^Department of Clinical Immunology, Tongji Hospital, Tongji Medical College, Huazhong University of Sciences and Technology, Wuhan, China; ^2^Department of Dermatology, Union Hospital, Tongji Medical College, Huazhong University of Science and Technology, Wuhan, China; ^3^Department of Biological Sciences, Faculty of Science,Engineering and Technology, Chuka University, Chuka 109-60400, Kenya; ^4^Department of Pediatrics & Child Health, The University of Zambia-University College London Medical School at Zambia, Lusaka, Zambia; ^5^Department of Dermatology, Union Shenzhen Hospital, Huazhong University of Science and Technology, Shenzhen 518052, China

## Abstract

*Neisseria gonorrhoeae* (*N. gonorrhoeae,* gonococci, or GC), the etiologic agent of gonorrhea, is a human-obligate bacterial pathogen. The GC surface contains pili that mediate the adherence to host cells. Studies have shown that GC pili, coded by pilin genes, undergo remarkable changes during human experimental gonorrhea, possibly generated by DNA phase variation during infection. The question that arises is whether the changes in pilins can alter the adherence capacity of *N. gonorrhoeae* to host cells. In this study, six variants initially isolated from male volunteers infected with one single clone of GC were examined for their adherence patterns with human Chang conjunctiva cells. In this study, we showed that the variants showed distinct adherence patterns to this cell line under light microscopy and scanning electron microscopy. Moreover, two reisolates showed higher adherence capacities than that of the input strain. The results provide an additional example as to how the pilus variation may play a role in the pathogenesis of *N. gonorrhoeae*.

## 1. Introduction

The Gram-negative bacterium *N. gonorrhoeae* (*N. gonorrhoeae,* gonococci, or GC) is the etiologic agent of sexually transmitted disease, gonorrhea, the second most common sexually transmitted infection after chlamydia infection [1]. Studies have suggested that GC has the ability to facilitate the human immunodeficiency virus infection [2–5]. With more than 106 million cases per year, the emergence of an antimicrobial resistance gonococcus (GC), especially a “Super Bug” GC [6–9], has become a worldwide concern [10, 11]. Without a highly effective vaccine to prevent gonorrhea, it presents a substantial challenge to public health [12, 13].

GC is an obligate human pathogen that possesses the tremendous ability to change its surface components, most notably the pili [14, 15], opacity-associated (Opa) proteins [16, 17], and lipooligosaccharides (LOSs) [18, 19]. It is expected that such a remarkable capacity to change may have a significant impact as an immune escape mechanism [20–22]. A series of very controversial human experiments were initially carried out by Swanson et al. to understand the roles of pili, Opa proteins, and LOSs in the pathogenesis of GC infection [15, 19, 23–25]. The results confirmed that all three major components varied significantly during the human experimental gonorrhea, indicating that these three major GC components are virulence factors [26–28].

The antigenic repertoire of GC surface pili is encoded by pilE and pilS genes [29]. However, the genes are subject to DNA phase and antigenic variations, which produce millions of different antigen variants during gonococcal infection [30, 31]. The *recA* gene controls the DNA phase variation in *N. gonorrhoeae*. As a result, in a *recA*-deficient strain, the phase variation by pili occurs at a 100–1000-fold reduced rate [32].

GC pili play an essential role in the mediation of adherence to hosts [33] and changes in the pilin amino acids may alter the adherent capacity of *N. gonorrhoeae* [23]. Studies by Swanson et al. showed that the pilin sequences of almost all of the reisolates were changed during infection. However, no studies on the biological functions were conducted with these reisolates. In this study, the adherence patterns of host-pathogen interaction were examined with scanning electron microscopy (SEM) in *N. gonorrhoeae* MS11_mk_ isolated from human experimental gonorrhea.

## 2. Materials and Methods

### 2.1. Ethics Statement

All experimental procedures were specifically approved for this study by the Medical Ethics Committee of Tongji Hospital and were conducted in accordance with institutional guidelines (IRB : TJC20140113). All volunteers involved in the experiment signed informed consent.

### 2.2. Bacterial Strains

The *N. gonorrhoeae* MS11_mk_ used in this study was reisolated from human experimental gonorrhea by Swanson et al. in the 1980s [23]. Briefly, two male volunteers were challenged with GC suspension and urine samples of the subjects were collected for the isolation of GC 2 days after inoculation. All strains were cultured and maintained as previously described [34]. The input GC are the pilus^+^(*P*^+^) and LOS_b_ phenotype and express no outer membrane protein II (Opa^−^) [19, 34]. Both the input and reisolated GC were constructed and designated to be recA mutants, following the methods by Koomey et al. [35]. A cosmid clone containing the *recA* gene of MS11 was isolated by complementation of the *E. coli* HB101 recA mutation. Defined mutations in and near the cloned gonococcal recA gene were constructed in vitro and concurrently associated with a selectable genetic marker for *N. gonorrhoeae* and the mutated alleles were then reintroduced into the gonococcal chromosome by transformation-mediated marker rescue [35]. All GC were grown overnight for 18 h on solid GC agar at 37°C in a humidified 5% CO_2_ atmosphere, unless otherwise stated.

### 2.3. Immunoblotting

GC was suspended in phosphate-buffered saline (PBS) to the same optical density at 540 nm and immersed in 20 ul solubilizing solution (containing 4% sodium dodecyl sulfate and 8% 2-mercaptoethanol). After heating in a boiling water bath for 10 min, 10 *μ*l of each lysate was applied to the sodium dodecyl sulfate-polyacrylamide gel electrophoresis (SDS-PAGE) slab gel. After electrophoresis, the SDS-PAGE-separated components were electrophoretically transferred to a sheet of nitrocellulose, which, after blocking with a solution containing bovine serum albumin, was incubated with rabbit monoclonal antiserum MCO2 for 2 h with a dilution of 1 : 500. The visualization of pilin antigen-bound antibodies through reaction with ^125^I-protein A was followed by autoradiography [28, 34]. The LOS was isolated from each of the strains, and approximately 1 mg of the LOS was analyzed on a 13% acrylamide gel. The LOS phenotypes were confirmed with rabbit anti-LOS MAbs (1 : 200) specific for LOSa and LOSb, and the primary antibodies were detected with a goat anti-rabbit IgG [36]. Opa expression was confirmed by SDS-PAGE and immunoblotting with monoclonal antibody 4B12 with a dilution of 1 : 200, which recognizes all Opa proteins from *N. gonorrhoeae* MS11_mk_ [37].

### 2.4. Adherence and Internalization Assays

The human conjunctiva cells, which were used for all adhesion and invasion assays, were cultured in RPMI 1640 medium (GIBCO/BRL) with 10% fetal calf serum (HyClone). For adherence assays, the cells were grown to confluence (≈2 × 10^5^ cells per well) in 24-well culture plates (Falcon) and washed twice with serum-free RPMI 1640. GC was suspended in RPMI 1640 at an OD_540_ of 0.04, and 0.5 ml of the bacterial suspensions was added to each well. The plates were incubated at 37°C in the presence of 5% CO_2_ in a humidified atmosphere for 1.5 to 6 h. Experiments were terminated by washing three times with 2 ml of serum-free RPMI 1640. Adherent bacteria were counted by suspending the cells in sterile PBS containing 0.5% saponin (Calbiochem) for 5 min at 37°C and plating dilutions on GC agar plates. The level of adherence was calculated by determining the CFU associated with the host cell monolayers after a 24 h incubation period. Internalization assays were similarly conducted like the adherence assays, but after the period of the bacterial interaction with cell lines, the monolayers were washed twice and then incubated for 90 min with 1.5 ml of RPMI 1640 supplemented with 100 *μ*g/ml gentamycin (GIBCO/BRL).

### 2.5. Scanning Electron Microscopy (SEM)

The microscopy assay followed the protocol by Stephens et al. with some modifications [38]. The infection process has been described above. After a coinoculation of 5 h at 37°C in the presence of 5% CO_2_ in a humidified atmosphere, the wells were moderately washed with filtered-sterilized PBS to remove the unbound bacteria. Thereafter, the culture was fixed in a 3% glutaraldehyde solution buffered to pH 7.3 with 0.1 M sodium cacodylate. After postfixation in 1% osmium tetroxide in the same buffer, samples were dehydrated in increasing concentrations of ethanol and = critical point dried with CO_2_. The samples were coated with gold-palladium before examination in a scanning microscope (JSM-35CF; JEOL USA, Peabody, MA), as described elsewhere in detail [39].

### 2.6. Normal Human Serum (NHS) and Serum Killing

Serum samples were collected intravenously from volunteers with no history of GC infection. The sera were aliquoted and stored at −70°C. The NHS was immediately thawed before use. The inactivated serum was achieved by heating the NHS at 56°C for 30 min. The method used to evaluate the serum sensitivity of bacterial strains was described previously [36]. The GC were propagated on GC HEPES (N-2-hydroxyethylpiperazine-N-2-ethanesulfonic acid)-agarose medium (GCHA) in case they formed into clusters. After culturing for 14 to 16 h, bacteria were suspended in PBS to an OD_540_ of 0.5 and diluted to 1 : 10 in RPMI medium. Diluted bacterial suspensions (50 *μ*l) were mixed with an equal volume of dilutions of 10% NHS in RPMI medium and incubated at 37°C in 5% CO_2_ for 60 min. Control assays were done in a manner that was identical to the heat-inactivated serum (56°C for 30 min). After performing serial 10-fold dilutions of the mixtures, the number of viable bacteria was determined by plating on GC agar. Serum resistance levels were determined by comparing the number of surviving bacteria treated with fresh NHS with that of the survivors treated with heat-inactivated NHS (defined as 100%).

### 2.7. Statistical Analyses

All statistical analyses were completed using Prism software, version 6 (Graph Pad, San Diego, CA, USA). Statistical significance was assessed using Student's *t*-test for the univariate analysis of two sets of data and two-way ANOVA was used for multiple comparisons; ^*∗∗∗*^*p* < 0.001.

## 3. Results

### 3.1. Construction of recA-Deficient Mutants

**Figure 1:**
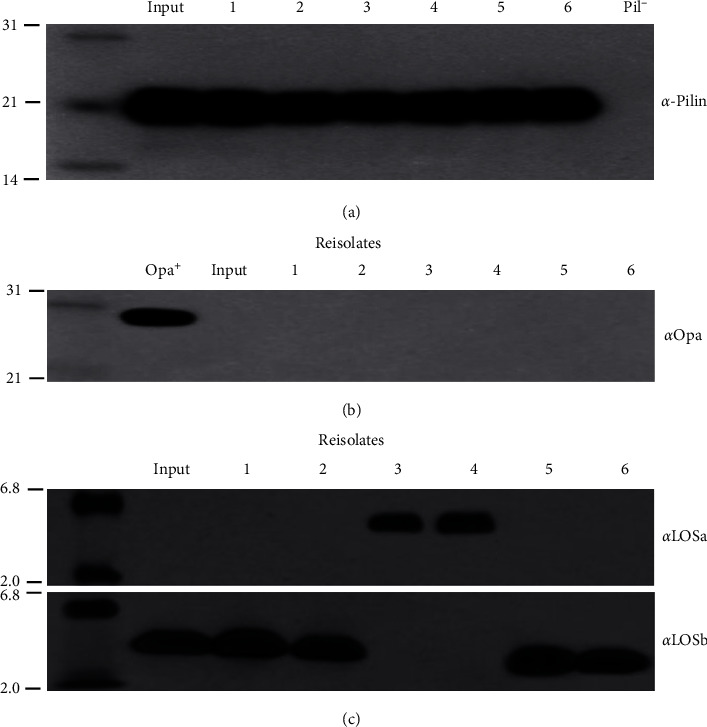
Analysis of the pili, Opa protein, and LOS phenotypes of reisolates. (a) Whole cell lysates of reisolates were subjected to SDS-PAGE and were immunoblotted with anti-pilus monoclonal antibody MCO2. (b) The expression of the Opa protein was detected with monoclonal antibody 4B12, which reacts with all of the Opa proteins expressed by the strain MS11mk. (c) The LOS preparations were analyzed by SDS-PAGE and then immunoblotting with *α*LOSa and *α*LOSb. Autoradiography was used to detect the LOS labelled in vitro. Lanes 1 to 6 represent G29, 4R1, SA, 7uB, 6uF, and V3, respectively. The molecular mass markers (in kilodaltons) are indicated on the left side of the panels.

The recA gene accounts for the generation of pilus antigenic diversity and piliation phase [32]. To reduce the rates of pili changes during *in vitro* experiments, both input and reisolated GC [23] were constructed to be recA mutants, following the methods by Koomey and Falkowet al. [35]. SDS-PAGE and immunoblotting were carried out to determine the phenotype of LOS, Opa protein, and pili of the reisolates and input strain (Figure 1). All of the 6 reisolates expressed pilin subunits that exhibited the same electrophoretic mobility as the input strain (Figure 1(a)), while previous studies by Swanson et al. indicated that the pilin mRNA sequencing of these strains exhibited multiple sequence changes in pilin mRNAs compared to the input GC [23]. The reisolates expressed no outer membrane protein (Opa) (Figure 1(b)). Interestingly, out of the six reisolates, two appeared to be LOSa phenotype (Figure 1(c)), which was probably generated from the parental strains that were LOSb phenotype. This phenomenon was in accordance with studies by other researchers [18, 19], as the GC LOS transition frequently occurs in vivo.

### 3.2. Reisolates Exhibit Distinct Adherence Patterns to Chang Conjunctiva Cells

**Figure 2:**
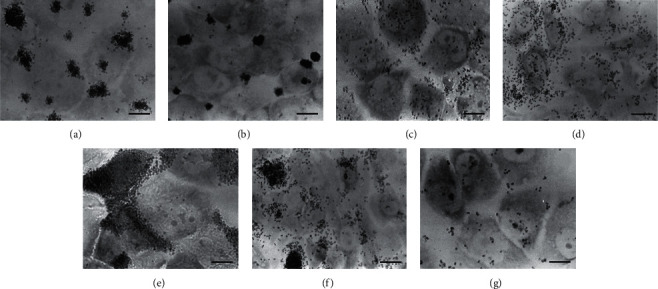
Light micrograph showing the patterns of adherence of reisolates to human conjunctiva cells ((a–g) represent MS11, G29, 4R1, SA, 7uB, 6uF, and V3, resp.). After the 5 h adherence assay, nonadherent bacteria were removed by washing three times with sterilized PBS. Cells were stained with a modified Wright stain and viewed under light microscopy. Scale bar: 10 *μ*m.

Light microscopy and SEM were used to examine the adherence patterns of the input strain and reisolates to Chang conjunctiva cells, which represent tissues that are natural sites for infection by GC [40, 41]. As illustrated in Figure 2, the reisolate G29, being identical to the input strain MS11, accumulated together to form clusters between the interface of bacteria and Chang cells (Figures 2(a) and 2(b)). The reisolates 4R1 and SA were noticeably distributed on the cell surfaces (Figures 2(c) and 2(d)), while 6uF possessed small parts that formed bacterial clusters similar to the MS11 strain (Figure 2(f)). The 7uB formed clusters to facilitate higher adherence to Chang cells as compared with the other reisolates (Figure 2(e)). For strain V3, only a few organisms were visualized on the cells, indicating that the V3 strain was less adherent than the other reisolates (Figure 2(g)).

**Figure 3:**
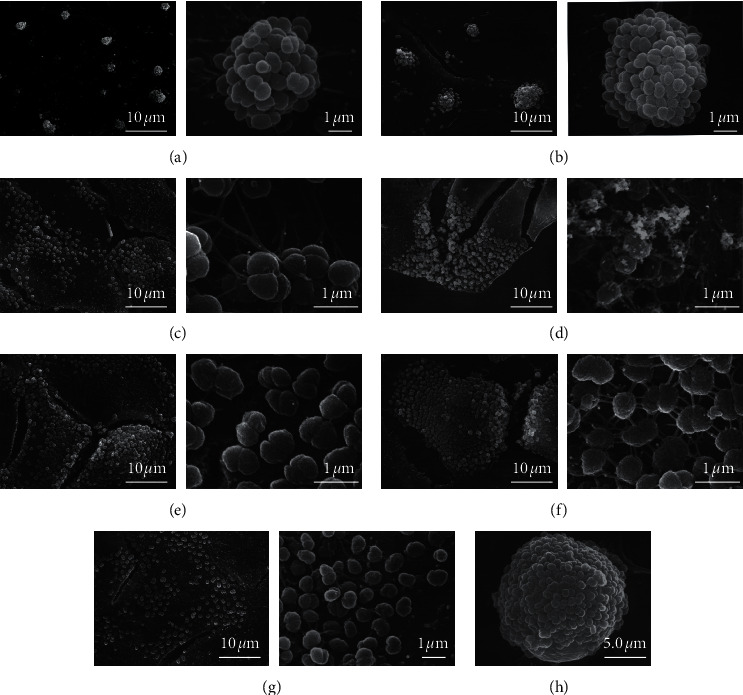
Scanning electron micrograph showing adherence of bacterial reisolates to host cells. Each panel depicts a representative group of bacteria ((a–h) represent MS11, G29, 4R1, SA, 7uB, 6uF, V3, and 7uB, resp.). The left panel indicates the low power magnification and the right panel indicates the higher magnification. Scale bars are mentioned at the right bottom of each image. Figure 3(h) depicts the interaction of 7uB to dust or unknown bead particles.

Under the SEM (Figure 3), the reisolate G29 formed morphologically similar ball-like bacterial clusters as the input strain MS11. This finding was consistent with observations of light microscopy (Figures 3(a) and 3(b)). Although the SA and 4R1 reisolates exhibited similarly constituted morphologies, SA appeared to establish more noticeably sufficient linkages with one another than 4R1 (Figures 3(c) and 3(d)). Furthermore, the 7uB displayed remarkable adherent junctions between the GC and conjunctiva cells including intra-GC, followed by 6uF, 4R1, and SA. The interactions between abundant pili of 7uB were able to promote the formation of a complex network on the surface of conjunctiva cells (Figure 3(e)). Moreover, the flattened 6uF was distinguishable from MS11 and 7uB (Figure 3(f)). However, the V3 showed relatively incompact connections and were distributed separately at the interface of the epithelial cells (Figure 3(g)). Interestingly, a spherical bacterial cluster distributed on the surface of an unknown material (beads or dust) was inadvertently observed. The strain 7uB was able to accumulate with tight conjunctions to form a golf ball-like structure (Figure 3(h)). The observations made with SEM were similar to the findings of light microscopy. Taken together, the results indicated that with regard to the adherence to host cells, the GC underwent tremendous modulations during experimental gonorrhea.

### 3.3. Reisolates Adhered to and Internalized by Chang Conjunctiva Cells

**Figure 4:**
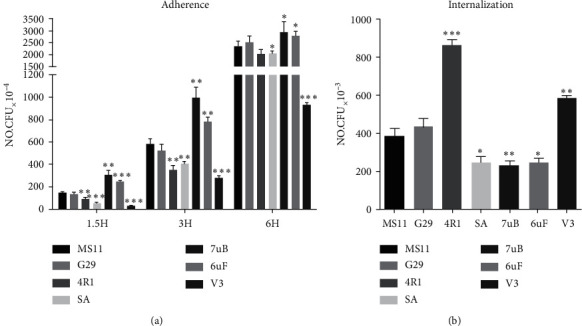
The (a) adherence and (b) internalization of reisolates and input strain (MS11) to human Chang conjunctiva cells. The level of adherence to cells was calculated by determining the cell-associated CFUs of the host cell monolayers (coincubation for 1.5 to 6 h after an incubation period of 24 h). For internalization, an approach similar to adherence assays by killing the extracellular bacteria with 100 *μ*g/ml (final concentration) gentamicin as previously described. The number of internalized bacteria was determined by counting CFUs recovered following gentamicin treatment. ^*∗∗∗*^*p* < 0.001. ^*∗∗*^*p* < 0.01. ^*∗*^*p* < 0.05.

The input strain MS11 and six reisolates with the Opa^−^ phenotype were incubated with human Chang conjunctiva cells. The reisolate 7uB exhibited higher adherence compared with the other strains, followed by 6uF, G29, MS11, and SA (Figure 4(a)). The V3 showed relatively lower adherence capacity as compared to the input strain (Figure 4(a)). G29 exhibited almost the same level of adherence with that of input strain MS11. It is demonstrated in Figure 4(a) that the adherence capacities of these bacteria increase with a prolonged period of incubation. The gentamycin protection assays for bacterial internalization revealed that the reisolate 4R1 significantly invaded the cells (Figure 4(b)). Interestingly, the 7uB, 6uF, and SA displayed moderately lower internalization levels, which were inconsistent with the findings of adherence. The disparity in the data of the adherence and internalization may be predominantly attributed to alterations in bacterial components and their varying degrees of functional aspects during host–pathogen interaction.

### 3.4. The Sensitivity of Reisolates to Serum Killing

**Figure 5:**
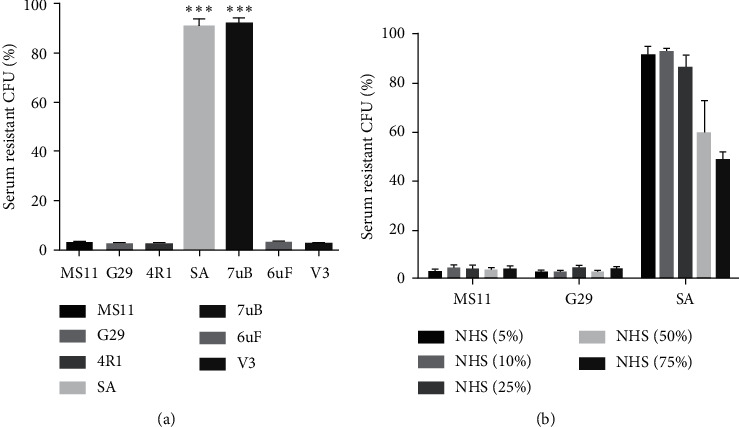
Serum resistance levels of the input and reisolated strains were assayed at different concentrations of the NHS. Bacterial suspensions were allowed to coincubate with the NHS for 60 min, and control assays were performed identically with heat-inactivated serum (56°C for 30 min). The serum resistance of the input strain and reisolates were assayed for their resistance levels at different concentrations of NHS (10% for (a); 5%–75% for (b)). All of the experiments were performed in triplicate. ^*∗∗∗*^*p* < 0.001.

Reisolates SA and 7uB exhibited markedly higher resistance to the effect of serum killing compared to the input strain MS11 and the remaining reisolates in the presence of 10% NHS (Figure 5(a)). The strains MS11, G29, and SA were further examined at different concentrations of NHS, and the sensitivity of SA attenuated by 50% upon incubation with 75% NHS (Figure 5(b)). The resistance to NHS results from the specific masking of the LOS epitopes that react with the nonsialylated molecules among bactericidal antibodies in human serum [42, 43]. The LOSb variants (which can be sialylated) were much more sensitive to the killing effect of NHS than the LOSa variants (which cannot be sialylated) [36]. All strains used in this study were Opa^−^ GC; the SA and 7uB were LOSa phenotype. This observation was identical to that of the previous study. These results indicated that the input GC retained various components that are involved in the bacterial resistance to serum killing during infection.

## 4. Discussion

*Neisseria gonorrhoeae* (*N. gonorrhoeae,* gonococci, or GC), the etiologic agent of gonorrhea, is a human-obligate bacterial pathogen, and therefore, there still is no animal model. Dr. John Swanson's group led studies by using human volunteers as the models to study the molecular pathogenesis of GC (human experimental gonorrhea) [23, 28, 32, 37, 44, 45]. One of the studies was to challenge human volunteers through the urethra with a single, live, and virulent clone of GC MS11_mk_. After showing signs of gonorrhea, the discharged GC were collected and the reisolates were stored for further studies. The major finding of this 1987 study was that the pilin sequences of almost all reisolates were changed during infection [23], indicating that pili play a major role during gonorrhea. Because no studies on biological functions were then conducted with these reisolates, the study was to understand in part the changes of these reisolates for the adherence to host cells.

“Seeing is believing.” This study focused on the distinct colony morphologies, adherent patterns of six GC reisolates from human experimental gonorrhea to Chang human conjunctiva cells. The results obtained from the presented study provide photographic adherent patterns of GC isolates to host cells, which are consistent with other reports, indicating the possibilities of surface component variations of GC during infection. As shown in Figure 1(a), both the input strain and reisolates expressed pilin. However, the pili of some strains are not visible when observed with SEM. This is consistent with the results reported by others [46]. Studies by Swanson et al. showed that pili exhibit highly variable characteristics both in the laboratory and in vivo through frequently programmed recombination events, including expression of different silent, partial pilin genes after their incorporation into the expressed complete pilin gene via gene conversion. Gonococcal pilus phase variation is involved in switches from piliation (*P*+) to nonpiliation (*P*−) and vice versa. The pilin gene recombination generates antigenically distinct pilins with variant epitopes, which can be detected by antibodies [23, 44, 47]. The recombination events might occur in the process of these experiments, generating nonpiliation (*P*−) GC strains where the pili are not visible.

The internalization of bacteria was not in accordance with adherence (Figure 4), and it should be noted that the levels of adherence and internalization were determined by counting the CFUs recovered from cell lysis. The SEM observations showed the GC tend to form clusters easily on epithelial cells. It was assumed that the bundled GC clusters might not be easily separated in the lysis buffers. Moreover, the input strain MS11 and reisolate G29 formed ball-like structures on the interface of epithelial cells. Such formation might block the contact between the GC and host cells, leading to poor internalization, while the V3 with loose colonies easily interacted with the host cells. The reisolate SA and 7uB, the most bundled strains, exhibited efficient resistance to serum killing (Figure 5) compared with parental MS11 and the other reisolates, which might be attributable to the LOS types. The bactericidal effect of the NHS results is mainly from the recognition and interaction between the bacteria-impelled antibody IgM and LOS, which elicits complement-mediated killing actions [48, 49]. The variation between the LOS types has been demonstrated in human experimental infections [19]. A previous study has illustrated that the LOSb variants were much more sensitive to the killing effect of NHS than the LOSa variants [36, 50]. Moreover, studies by Ward et al. suggested that *N. gonorrhoeae* harvested directly from a urethral exudate displayed unstable serum resistance and in vivo resistant strains may shift to sensitive ones when subcultured in a laboratory [51].

The 1987 study uncovered the possible pathogenic roles of gonococcal pili [23, 28, 32, 37, 44, 45]. Since then, progress in our understanding of gonococcal pilus-mediated pathogenesis and other pathogens with this model has been made. 1. The study has given an understanding of why GC is a human-obligate pathogen. This conclusion was based on the identification of pilC and its relationship with pilE. It appeared that the pilC piled on the tip of pilE mediates the human-specific interactions [52–54]. Furthermore, the CD46, a human-specific receptor, may also participate in the pilus-host interactions [55]. 2. The same approaches from the 1987 study have been applied in studying the other components such as Opa and LOS of GC, indicating that the human experimental gonorrhea has proven to be a very valuable tool to study the pathogenesis of gonorrhea [23, 28, 32, 37, 44, 45] and other pathogens. *Haemophilus ducreyi*, a Gram-negative pathogen and the cause of the sexually transmitted disease chancroid, was also injected into human skins to mimic infection processes of chancroid in later nineties by Dr. Spinola's Group [56, 57]. 3. They demonstrated that the main biological function of pili is to mediate host–pathogen interactions. The mouse model developed by Jerse's et al. group showed that they were still able to be infected by pilus negative GC, when the GC was coexpressed with other adherent molecules such as the Opa proteins [58, 59].

In summary, there are still no promising animal models, which fully mimic the pathogenic processes during GC infection. On the horizon, the results generated from the 1987 study on human experimental gonorrhea were the only way to ascertain the representative of pilus pathogenesis for gonorrhea. This enclosed study provides additional data as to why different pilus variants affect the pathogenesis for experimental gonorrhea. Knowledge of this may help in the development of an effective gonococcal vaccine.

## Data Availability

The experimental data used to support the findings of this study are included in the article.

## References

[1] Newman L., Rowley J., Vander Hoorn S. (2015). Global estimates of the prevalence and incidence of four curable sexually transmitted infections in 2012 based on systematic review and global reporting. *PLoS One*.

[2] Laga M., Manoka A., Kivuvu M. (1993). Non-ulcerative sexually transmitted diseases as risk factors for HIV-1 transmission in women. *AIDS*.

[3] Fleming D. T., Wasserheit J. N. (1999). From epidemiological synergy to public health policy and practice: the contribution of other sexually transmitted diseases to sexual transmission of hiv infection. *Sexually Transmitted Infections*.

[4] Ding J., Rapista A., Teleshova N. (2010). Neisseria gonorrhoeaeEnhances HIV-1 infection of primary resting CD^4+^ T cells through TLR2 activation. *The Journal of Immunology*.

[5] Zhang J., Li G., Bafica A. (2005). Neisseria gonorrhoeaeEnhances infection of dendritic cells by HIV type 1. *The Journal of Immunology*.

[6] Unemo M., Golparian D., Hellmark B. (2014). First three Neisseria gonorrhoeae isolates with high-level resistance to azithromycin in Sweden: a threat to currently available dual-antimicrobial regimens for treatment of gonorrhea?. *Antimicrobial Agents and Chemotherapy*.

[7] Shimuta K., Unemo M., Nakayama S. (2013). Antimicrobial resistance and molecular typing of *Neisseria gonorrhoeae* isolates in kyoto and Osaka, Japan, 2010 to 2012: intensified surveillance after identification of the first strain (H041) with high-level ceftriaxone resistance. *Antimicrobial Agents and Chemotherapy*.

[8] Unemo M., Golparian D., Nicholas R. (2012). High-level cefixime-and ceftriaxone-resistant Neisseria gonorrhoeae in France: NovelpenAMosaic allele in a successful international clone causes treatment failure. *Antimicrobial Agents and Chemotherapy*.

[9] Ohnishi M., Golparian D., Shimuta K. (2011). Is *Neisseria gonorrhoeae* initiating a future era of untreatable gonorrhea?: detailed characterization of the first strain with high-level resistance to ceftriaxone. *Antimicrobial Agents and Chemotherapy*.

[10] Unemo M., Del Rio C., Shafer W. M. (2016). Antimicrobial resistance expressed by *Neisseria gonorrhoeae*: a major global public health problem in the 21st century. *Microbiology Spectrum*.

[11] Costa-Lourenço A. P. R. d., Barros Dos Santos K. T., Moreira B. M., Fracalanzza S. E. L., Bonelli R. R. (2017). Antimicrobial resistance in Neisseria gonorrhoeae : history, molecular mechanisms and epidemiological aspects of an emerging global threat. *Brazilian Journal of Microbiology*.

[12] Kirkcaldy R. D., Harvey A., Papp J. R. (2016). Neisseria gonorrhoeaeAntimicrobial susceptibility surveillance-the gonococcal isolate surveillance project, 27 sites, United States, 2014. *MMWR. Surveillance Summaries*.

[13] Edwards J. L., Jennings M. P., Apicella M. A., Seib K. L. (2016). Is gonococcal disease preventable? the importance of understanding immunity and pathogenesis in vaccine development. *Critical Reviews in Microbiology*.

[14] Hagblom P., Segal E., Billyard E., So M. (1985). Intragenic recombination leads to pilus antigenic variation in *Neisseria gonorrhoeae*. *Nature*.

[15] Seifert H. S., Wright C. J., Jerse A. E., Cohen M. S., Cannon J. G. (1994). Multiple gonococcal pilin antigenic variants are produced during experimental human infections. *Journal of Clinical Investigation*.

[16] Sparling P. F., Cannon J. G., So M. (1986). Phase and antigenic variation of pili and outer membrane protein ii of *Neisseria gonorrhoeae*. *Journal of Infectious Diseases*.

[17] Stern A., Brown M., Nickel P., Meyer T. F. (1986). Opacity genes in *Neisseria gonorrhoeae*: control of phase and antigenic variation. *Cell*.

[18] Apicella M. A., Shero M., Jarvis G. A. (1987). Phenotypic variation in epitope expression of the *Neisseria gonorrhoeae* lipooligosaccharide. *Infection and Immunity*.

[19] Schneider H., Griffiss J. M., Boslego J. W. (1991). Expression of paragloboside-like lipooligosaccharides may be a necessary component of gonococcal pathogenesis in men. *Journal of Experimental Medicine*.

[20] Hedges S. R., Mayo M. S., Mestecky J., Hook E. W., Russell M. W. (1999). Limited local and systemic antibody responses toNeisseria gonorrhoeae during uncomplicated genital infections. *Infection and Immunity*.

[21] Meyer T. F., Gibbs C. P., Haas R. (1990). Variation and control of protein expression in Neisseria. *Annual Review of Microbiology*.

[22] Van Putten J. P. (1993). Phase variation of lipopolysaccharide directs interconversion of invasive and immuno-resistant phenotypes of *Neisseria gonorrhoeae*. *The EMBO Journal*.

[23] Swanson J., Robbins K., Barrera O. (1987). Gonococcal pilin variants in experimental gonorrhea. *Journal of Experimental Medicine*.

[24] Jerse A. E., Cohen M. S., Drown P. M. (1994). Multiple gonococcal opacity proteins are expressed during experimental urethral infection in the male. *Journal of Experimental Medicine*.

[25] Ramsey K. H., Schneider H., Cross A. S. (1995). Inflammatory cytokines produced in response to experimental human gonorrhea. *Journal of Infectious Diseases*.

[26] Schoolnik G. K., Buchanan T. M., Holmes K. K. (1976). Gonococci causing disseminated gonococcal infection are resistant to the bactericidal action of normal human sera. *Journal of Clinical Investigation*.

[27] Swanson J., Gotschlich E. C. (1973). Electron microscopic studies on streptococci. *Journal of Experimental Medicine*.

[28] Swanson J., Mayer L. W., Tam M. R. (1982). Antigenicity of *Neisseria gonorrhoeae* outer membrane protein(S) iii detected by immunoprecipitation and western blot transfer with a monoclonal antibody. *Infection and Immunity*.

[29] Meyer T. F., Billyard E., Haas R., Storzbach S., So M. (1984). Pilus genes of *Neisseria gonorrheae*: chromosomal Organization and DNA sequence. *Proceedings of the National Academy of Sciences*.

[30] Haas R., Meyer T. F. (1986). The repertoire of silent pilus genes in *Neisseria gonorrhoeae*: evidence for gene conversion. *Cell*.

[31] Swanson J., Bergstrom S., Robbins K. (1986). Gene conversion involving the pilin structural gene correlates with pilus+ ⇄ pilus− changes in Neisseria gonorrhoeae. *Cell*.

[32] Koomey M., Gotschlich E. C., Robbins K., Bergstrom S., Swanson J. (1987). Effects of reca mutations on pilus antigenic variation and phase transitions in *Neisseria gonorrhoeae*. *Genetics*.

[33] Punsalang A. P., Sawyer W. D. (1973). Role of pili in the virulence of *Neisseria gonorrhoeae*. *Infection and Immunity*.

[34] Swanson J., Barrera O. (1983). Gonococcal pilus subunit size heterogeneity correlates with transitions in colony piliation phenotype, not with changes in colony opacity. *Journal of Experimental Medicine*.

[35] Koomey J. M., Falkow S. (1987). Cloning of the reca gene of *Neisseria gonorrhoeae* and construction of gonococcal reca mutants. *Journal of Bacteriology*.

[36] Chen T., Swanson J., Wilson J., Belland R. J. (1995). Heparin protects Opa+ Neisseria gonorrhoeae from the bactericidal action of normal human serum. *Infection and Immunity*.

[37] Swanson J., Barrera O., Sola J., Boslego J. (1988). Expression of outer membrane protein ii by *gonococci* in experimental gonorrhea. *Journal of Experimental Medicine*.

[38] Stephens D. S., Hoffman L. H., Mcgee Z. A. (1983). Interaction of *Neisseria meningitidis* with human nasopharyngeal mucosa: attachment and entry into columnar epithelial cells. *Journal of Infectious Diseases*.

[39] Hitchcock P. J., Brown T. M., Corwin D. (1985). Morphology of three strains of contagious equine metritis organism. *Infection and Immunity*.

[40] Kupsch E. M., Knepper B., Kuroki T., Heuer I., Meyer T. F. (1993). Variable opacity (Opa) outer membrane proteins account for the cell tropisms displayed by *Neisseria gonorrhoeae* for human leukocytes and epithelial cells. *The EMBO Journal*.

[41] Makino S., Van Putten J. P., Meyer T. F. (1991). Phase variation of the opacity outer membrane protein controls invasion by *Neisseria gonorrhoeae* into human epithelial cells. *The EMBO Journal*.

[42] Parsons N. J., Andrade J. R. C., Patel P. V., Cole J. A., Smith H. (1989). Sialylation of lipopolysaccharide and loss of absorption of bactericidal antibody during conversion of gonococci to serum resistance by cytidine 5′-monophospho-N-acetyl neuraminic acid. *Microbial Pathogenesis*.

[43] Parsons N. J., Patel P. V., Tan E. L. (1988). Cytidine 5′-monophospho-N-acetyl neuraminic acid and a low molecular weight factor from human blood cells induce lipopolysaccharide alteration in gonococci when conferring resistance to killing by human serum. *Microbial Pathogenesis*.

[44] Swanson J., Robbins K., Barrera O., Koomey J. M. (1987). Gene conversion variations generate structurally distinct pilin polypeptides in *Neisseria gonorrhoeae*. *Journal of Experimental Medicine*.

[45] Swanson J. (1973). Studies on gonococcus infection. *Journal of Experimental Medicine*.

[46] Griffiss J. M., Lammel C. J., Wang J., Dekker N. P., Brooks G. F. (1999). *Neisseria gonorrhoeae* coordinately uses pili and Opa to activate hec-1-B cell microvilli, which causes engulfment of the gonococci. *Infection and Immunity*.

[47] Swanson J., Bergström S., Barrera O., Robbins K., Corwin D. (1985). Pilus- gonococcal variants. Evidence for multiple forms of piliation control. *Journal of Experimental Medicine*.

[48] Paz H. d. l., Cooke S. J., Heckels J. E. (1995). Effect of sialylation of lipopolysaccharide of *Neisseria gonorrhoeae* on recognition and complement-mediated killing by monoclonal antibodies directed against different outer-membrane antigens. *Microbiology*.

[49] Apicella M. A., Westerink M. A., Morse S. A. (1986). Bactericidal antibody response of normal human serum to the lipooligosaccharide of Neisseria gonorrhoeae. *Journal of Infectious Diseases*.

[50] Tan E. L., Patel P. V., Parsons N. J., Martin P. M., Smith H. (1986). Lipopolysaccharide alteration is associated with induced resistance of *Neisseria gonorrhoeae* to killing by human serum. *Microbiology*.

[51] Ward M. E., Watt P. J., Glynn A. A. (1970). *Gonococci* in urethral exudates possess a virulence factor lost on subculture. *Nature*.

[52] Seifert H. S., Ajioka R. S., Marchal C., Sparling P. F., So M. (1988). DNA transformation leads to pilin antigenic variation in *Neisseria gonorrhoeae*. *Nature*.

[53] Rudel T., Scheuerpflug I., Meyer T. F. (1995). *Neisseria* pilc protein identified as type-4 pilus tip-located adhesin. *Nature*.

[54] Scheuerpflug I., Rudel T., Ryll R., Pandit J., Meyer T. F. (1999). Roles of pilc and pile proteins in pilus-mediated adherence of *Neisseria gonorrhoeae* and *Neisseria meningitidis* to human erythrocytes and endothelial and epithelial cells. *Infection and Immunity*.

[55] Johansson L., Rytkonen A., Bergman P. (2003). Cd46 in meningococcal disease. *Science*.

[56] Brothwell J. A., Griesenauer B., Chen L., Spinola S. M. (2020). Interactions of the skin pathogen *Haemophilus ducreyi* with the human host. *Frontiers in Immunology*.

[57] Al-Tawfiq J. A., Bauer M. E., Fortney K. R. (2000). A pilus-deficient mutant of *Haemophilus ducreyi* is virulent in the human model of experimental infection. *Journal of Infectious Diseases*.

[58] Wu H., Jerse A. E. (2006). *α*-2,3-Sialyltransferase enhances Neisseria gonorrhoeae survival during experimental murine genital tract infection. *Infection and Immunity*.

[59] Soler-García A. A., Jerse A. E. (2007). *Neisseria gonorrhoeae* catalase is not required for experimental genital tract infection despite the induction of a localized neutrophil response. *Infection and Immunity*.

